# Association of alcohol types, coffee and tea intake with mortality: prospective cohort study of UK Biobank participants

**DOI:** 10.1017/S000711452200040X

**Published:** 2023-01-14

**Authors:** Sylva M. Schaefer, Anna Kaiser, Inken Behrendt, Gerrit Eichner, Mathias Fasshauer

**Affiliations:** 1 Institute of Nutritional Science, Justus-Liebig University of Giessen, Giessen 35390, Germany; 2 Mathematical Institute, Justus-Liebig University of Giessen, Giessen 35392, Germany; 3 Department of Internal Medicine (Endocrinology, Nephrology, and Rheumatology), University of Leipzig, Leipzig 04103, Germany

**Keywords:** Alcohol, Coffee, Metabolic syndrome, Mortality, Tea, UK Biobank, Wine

## Abstract

The present study examines how alcohol intake from wine and non-wine alcoholic beverages (non-wine) in g/d, as well as cups of coffee and tea included as continuous covariates and mutually adjusted are associated with all-cause, cancer, non-cancer and CVD mortality. Consumption was assessed in 354 386 participants of the UK Biobank cohort who drank alcohol at least occasionally and survived at least 2 years after baseline with 20 201 deaths occurring over 4·2 million person-years. Hazard ratios (HR) for mortality were assessed with Cox proportional hazard regression models and beverage intake fitted as penalised cubic splines. A significant U-shaped association was detected between wine consumption and all-cause, non-cancer and CVD mortality. Wine consumption with lowest risk of death (nadir) ranged from 19 to 23 g alcohol/d in all participants and both sexes separately. In contrast, non-wine intake was significantly and positively associated in a dose-dependent manner with all mortality types studied except for CVD in females and with the nadir between 0 and 12 g alcohol/d. In all participants, the nadir for all-cause mortality was 2 cups coffee/d with non-coffee drinkers showing a slightly increased risk of death. Tea consumption was significantly and negatively associated with all mortality types in both sexes. Taken together, light to moderate consumption of wine but not non-wine is associated with decreased all-cause and non-cancer mortality. A minor negative association of coffee consumption with mortality cannot be excluded whereas tea intake is associated with a consistently decreased risk of all mortality types studied.

Besides smoking and a sedentary lifestyle, unhealthy eating patterns are major contributors to morbidity, as well as all-cause and cause-specific mortality^(1,2)^. Various beverages including sugar- and artificially sweetened beverages have been implicated to contribute to metabolic and CVD, as well as cancer^(3,4)^. In contrast, for alcoholic drinks, coffee and tea, the association between intake and health effects is less clear.

Alcohol consumption is typically quantified in g/d and the extent of alcohol intake is frequently divided into categories, including no, light, moderate and heavy drinking. Restricted alcohol intake <16 g/d is recommended for both sexes by the National Health Service to keep health risks low^(5)^. Chronic light to moderate drinking was associated with decreased risks of incident type 2 diabetes mellitus^(6)^, myocardial infarction and stroke^(7)^ but increased risks of hypertension in men^(8)^ and liver cirrhosis in women^(9)^. Moderate alcohol drinking was linked with an increased risk for oral/pharynx, oesophageal squamous cell, colorectal, liver, female breast cancer and malignant melanoma on one hand and a decreased risk for kidney, thyroid and haematologic malignancy on the other hand^(10)^. Based on these findings, light to moderate alcohol consumption is regarded as safe by the National Health Service; however, it is not recommended to start drinking alcohol or to drink more frequently to gain potential health benefits^(5)^. Several studies suggest that differences exist between wine and non-wine alcoholic beverages (non-wine) concerning their associations with incident diabetes mellitus^(11)^, obesity^(12)^ and cancer^(13)^.

Coffee intake is often quantified in cups/d with moderate coffee consumption defined as 3–5 cups/d^(14)^. Moderate coffee consumption was associated with decreased risks of incident type 2 diabetes mellitus^(15)^, obesity^(16)^, CVD^(17)^, as well as liver and endometrial cancer^(18)^. Based on these results, moderate coffee consumption is considered safe^(19)^. Similar to coffee, moderate tea consumption is associated with decreased morbidity from type 2 diabetes mellitus^(20)^, ischaemic heart disease^(21)^, as well as several cancer subtypes^(22)^. Therefore, tea drinking is considered safe, but no specific guidelines exist concerning optimal intake.

Besides studies on the associations of alcohol, coffee and tea intake with morbidity, published evidence suggests that all-cause and cause-specific mortality are dose-dependently associated with the three beverages^(23–30)^. However, only few studies have assessed the association between wine *v*. non-wine consumption and mortality. Furthermore, models often include wine, non-wine, coffee and tea consumption as either linear predictors or even discretised ordinal predictors and mutual adjustments of the four beverages are not regularly performed. To address these limitations in the present study, associations between wine, non-wine, coffee and tea intake quantified on continuous scales and all-cause, as well as cause-specific, mortality hazards are determined in a large, well-characterised population of 354 386 UK Biobank participants using penalised cubic splines to allow, in particular, non-linear predictor effects. We hypothesised that non-linear relationships exist between the four beverage types and risk of death, as well as that intake levels linked to lowest mortality hazards depend on cause of death.

## Methods

### Study and participants

The study design of the multicentre, prospective UK Biobank cohort is described in detail at https://www.ukbiobank.ac.uk ^(31)^. In brief, more than 500 000 participants were recruited between 2006 and 2010 at twenty-two assessment centres across the UK with age at enrolment ranging from 38 to 73 years. At baseline, all participants were assessed by a self-completed touchscreen questionnaire, a personal interview and physical measurements as recently described^(32)^. The following five exclusion criteria (ec) were applied to all primary and sensitivity analyses: (1) missing smoking status; (2) missing socio-economic factors (i.e. ethnic background and/or overall health rating); (3) missing percentage body fat; (4) either missing information on beverage intake or being in the upper 0·1 % of alcohol, coffee or tea intake and (5) participants lost to follow-up or dying within 2 years after baseline (landmark analysis). All primary analyses with all outcome measures were performed in a primary cohort in which all non-alcohol drinkers were excluded in addition to ec1 to ec5 to remove all participants who might not drink alcohol due to health reasons (primary cohort; *n* 354 386; online Supplementary Fig. 1). Non-alcohol drinkers were defined as participants with a present alcohol intake of 0 g/d. This group consisted of never drinkers, that is, participants never drinking alcohol during their lifetime, and former drinkers, that is, participants drinking alcohol in the past but not in the present. In addition to the primary analyses, two sets of sensitivity analyses (cohorts S1 and S2) were run. In the first set, former drinker bias was controlled for by excluding former drinkers but not never drinkers in addition to ec1–ec5 (cohort S1; *n* 374 697). Since former drinkers include ex-drinkers who quit alcohol due to poor health, former drinker bias contributes to an apparently lower mortality of moderate drinkers^(33,34)^. In the second set of sensitivity analyses, only ec1–ec5 were applied, that is, non-drinkers were not excluded (cohort S2; *n* 399 866). Therefore, this set of analyses did not control for health issues in participants not drinking alcohol. The UK Biobank study was approved by the North West Multicentre Research Ethics Committee and all participants provided written informed consent before inclusion^(31)^.

### Exposure assessment

Estimation of alcohol from wine and non-wine, as well as coffee, and tea intake was performed similar as described by Bradbury and co-workers^(35)^. In brief, participants were asked about their weekly or monthly consumption of different alcoholic drinks during the baseline visit. Consumption of red wine and champagne plus white wine was included in the present analysis as wine intake whereas all other categories of alcoholic drinks, that is, beer plus cider, spirits, fortified wine and other alcoholic drinks, were included as non-wine. All alcoholic drinks were assumed to contain 10 g alcohol per portion except a pint of beer which was supposed to contain 20 g alcohol. Total weekly and monthly consumption of wine and non-wine was summed up for each participant. For an estimation of wine and non-wine intake in g/d, weekly and monthly consumption was divided by 7 and 30·4375, respectively. Furthermore, participants documented coffee and tea consumption as cups/d. An amount of 0·5 cups/d was assumed if ‘less than one’ cup of coffee or tea was recorded. Participants who did not indicate the extent of alcohol, coffee or tea intake including those answering ‘do not know’ or ‘prefer not to answer’ were excluded from the present analysis. In a subcohort of UK Biobank participants, only ‘consumption of other alcoholic drinks’ was not available. In these cases, this category was set to 0 g similar to Bradbury and co-workers^(35)^.

### Outcome assessment

Mortality data with date and underlying primary cause of death were provided by the National Health Service Information Centre for participants from England and Wales and by the National Health Service Central Register, Scotland for participants from Scotland^(36)^. Follow-up time was calculated between the date of baseline assessment and date of death or censoring (i.e. 23 March 2021), whichever came first. Besides overall mortality, the following two main mortality categories were defined according to the *International Statistical Classification of Diseases and Related Health Problems, Tenth Revision* (ICD-10) codes: cancer (C00-D48) and non-cancer (all but C00-D48). Furthermore, CVD (I00-I79) mortality was analysed. All analyses were performed in all participants, as well as in females and males separately.

### Statistical analyses

**Fig. 1.**
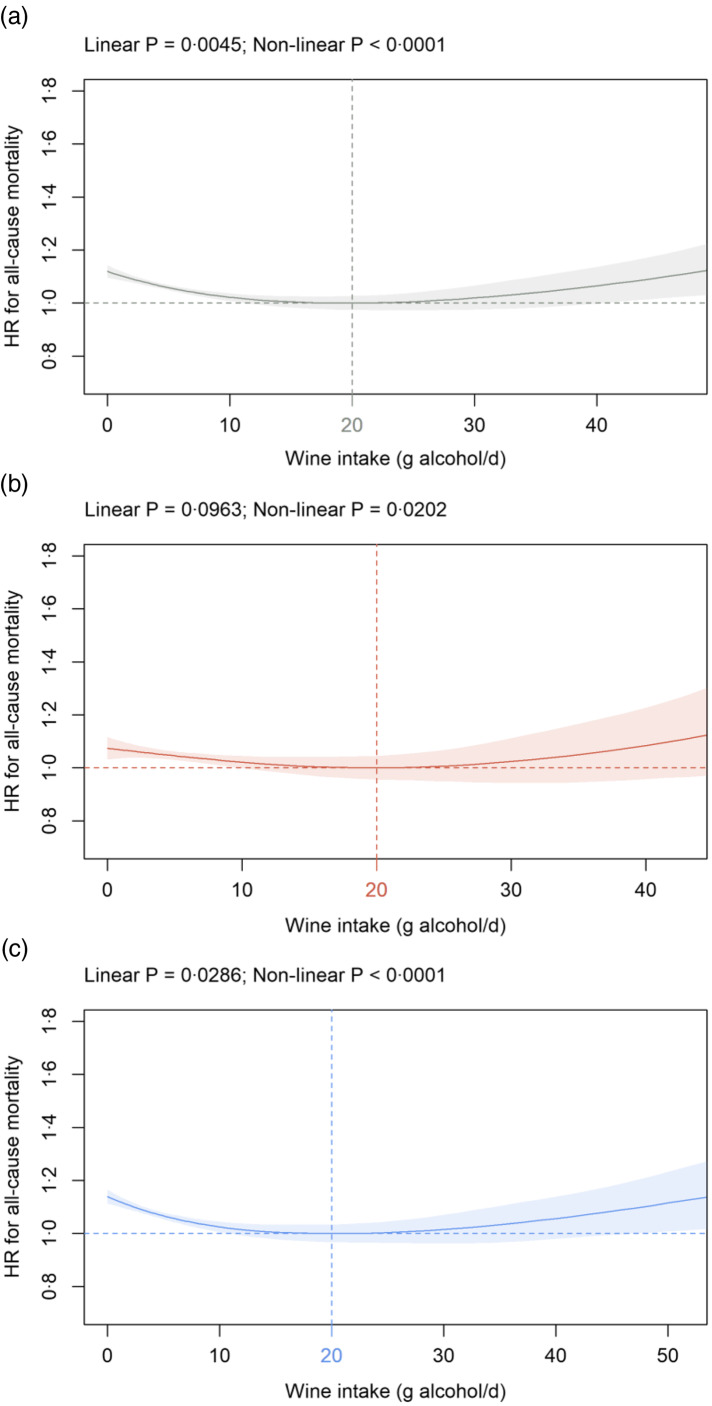
Association of wine intake (g alcohol/d) in: (a) all participants; (b) females and (c) males with all-cause mortality in the primary cohort. Data are adjusted for sex (all participants only), age, AHI, ethnicity, OHR, PA, percentage body fat and smoking status. Additionally, wine, non-wine, coffee and tea intake are mutually adjusted (e.g. wine intake is additionally adjusted for non-wine, coffee and tea intake) as summarised in the Methods section. Covariates not fulfilling the proportional hazard assumption (all participants: age; females: age; males: age, OHR, percentage body fat) are stratified. The nadir is indicated in grey (total cohort), red (female) and blue (male). HR: hazard ratio; AHI, annual household income; OHR, overall health rating; PA, physical activity.

**Fig. 2.**
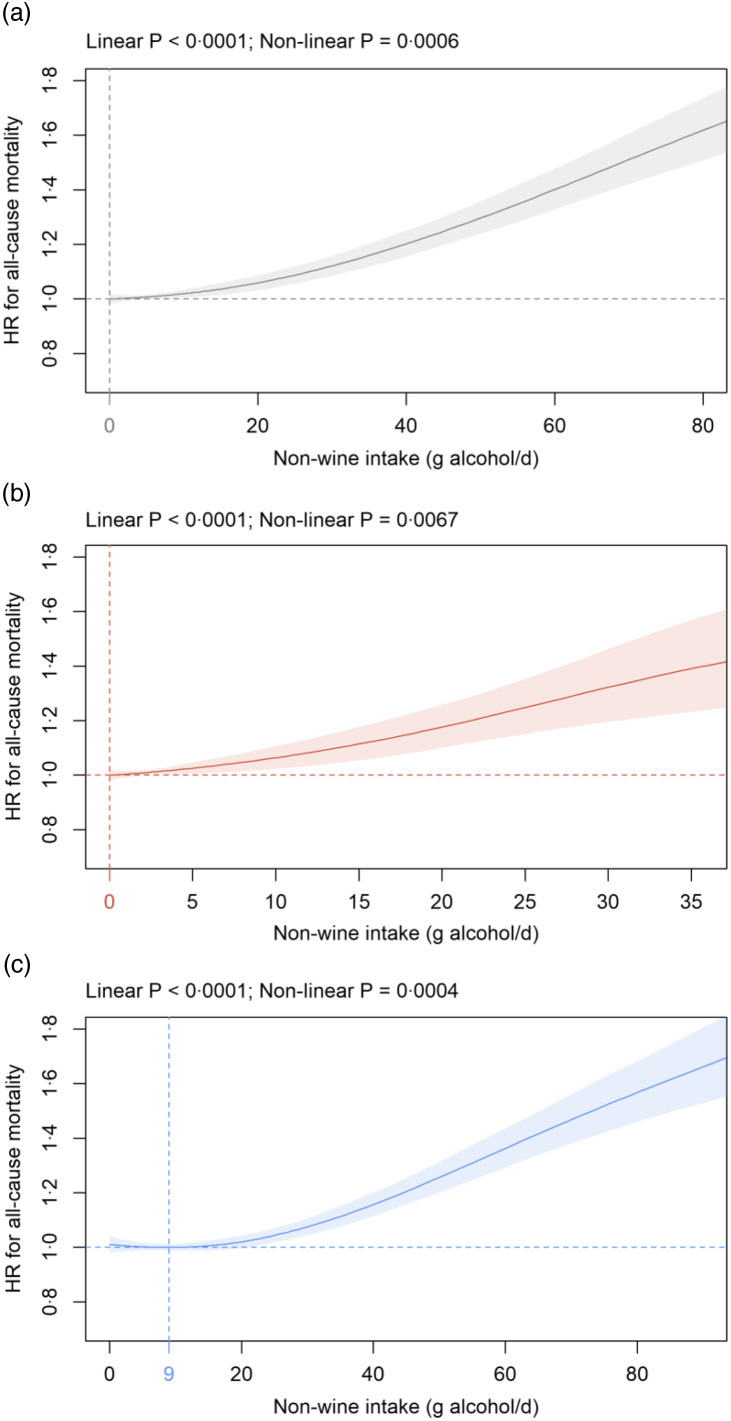
Association of non-wine intake (g alcohol/d) in: (a) all participants; (b) females and (c) males with all-cause mortality in the primary cohort. Data are adjusted and presented as indicated in Fig. 1.

**Fig. 3.**
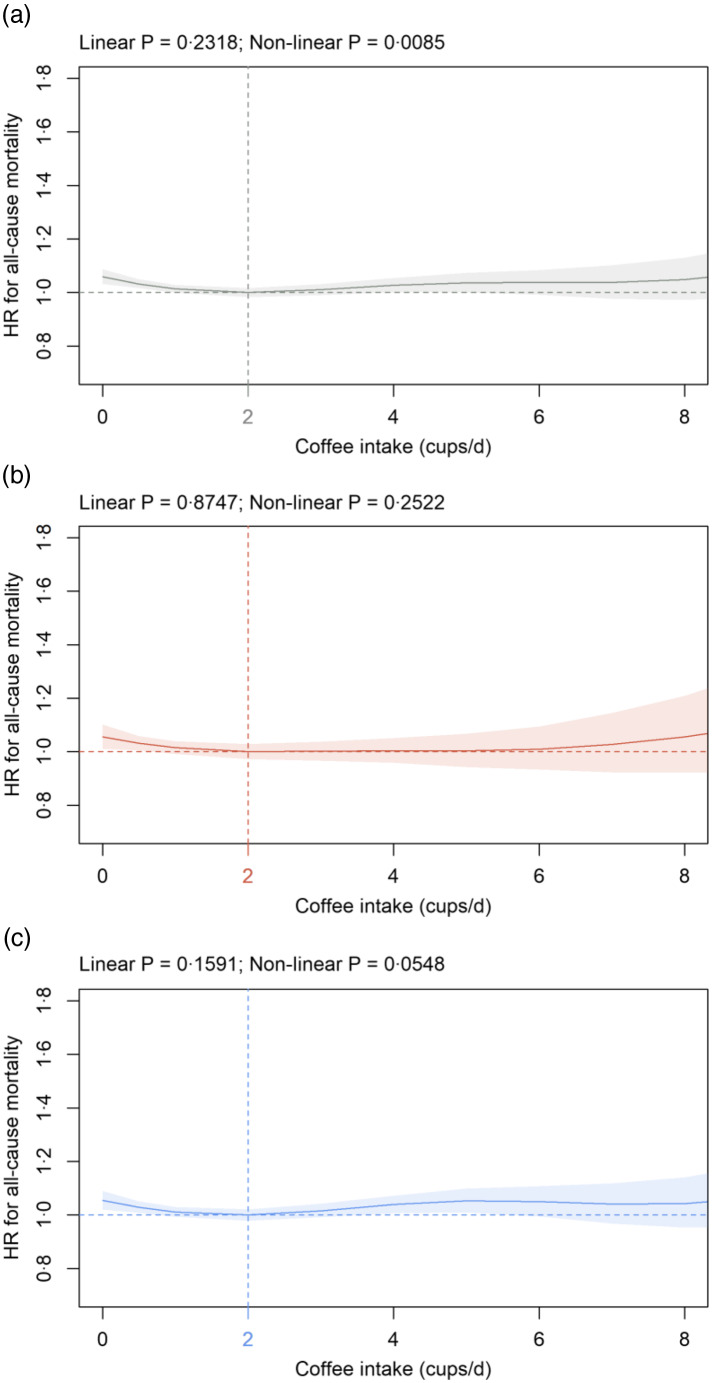
Association of coffee intake (cups/d) in: (a) all participants; (b) females and (c) males with all-cause mortality in the primary cohort. Data are adjusted and presented as indicated in Fig. 1.

**Fig. 4.**
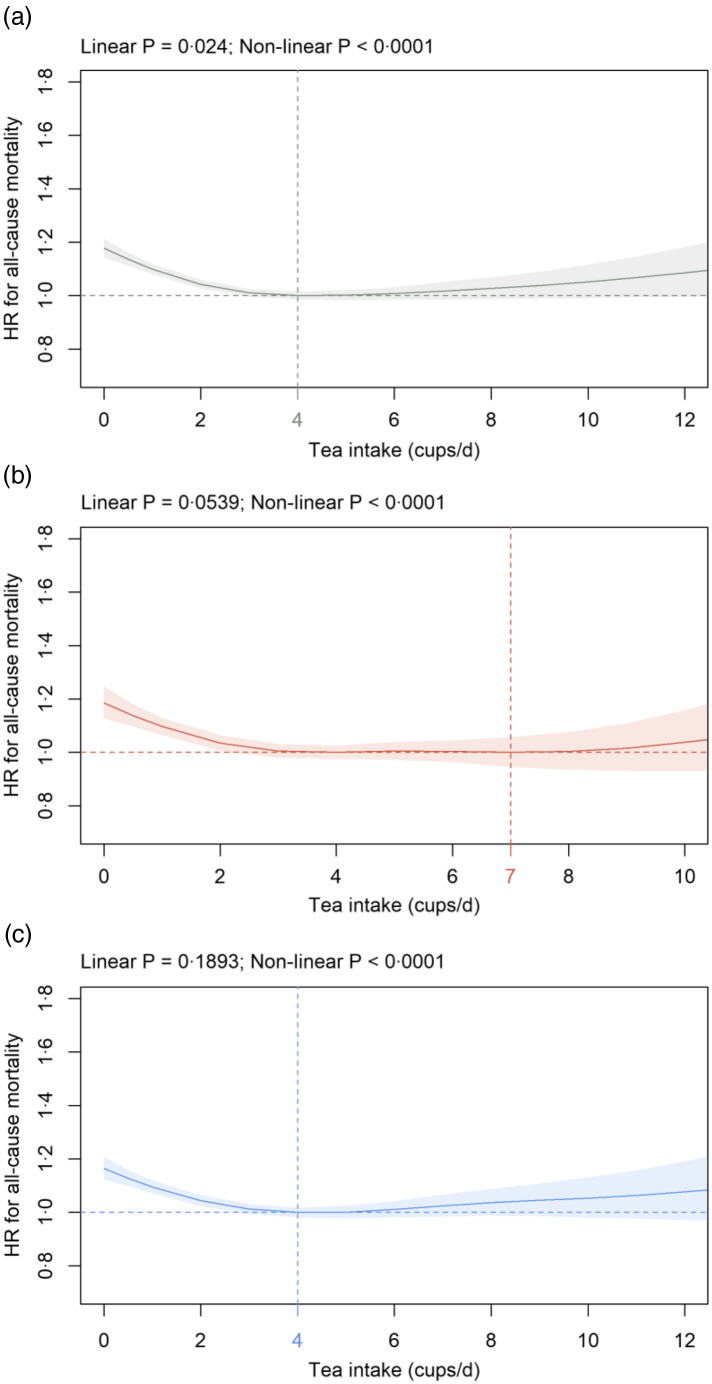
Association of tea intake (cups/d) in: (a) all participants; (b) females and (c) males with all-cause mortality in the primary cohort. Data are adjusted and presented as indicated in Fig. 1.

Data were imported, processed, analysed and graphically displayed with R version 4.0.5^(37)^ in combination with the packages readxl^(38)^, tidyverse^(39)^, venn^(40)^, skimr^(41)^ and survival^(42)^. Cox proportional hazard regression models were fitted with wine, non-wine, coffee and tea mutually adjusted and included as penalised cubic splines with their degrees of freedom set to 4. The analysis of each penalised cubic spline is segregated into its linear and non-linear effects whose significances are documented by the respective *P* values (p^lin^ for the linear and p^non-lin^ for the non-linear effect) of Wald-type tests for joint significance of the multiple coefficients associated with the respective linear or non-linear portion of the penalised spline fit^(43,44)^. These *P* values for the association of wine, non-wine, coffee and tea with mortality are depicted in Fig. 1–4, online Supplementary Fig. 2 and 3 and Supplementary Tables 2–4. In all analyses, the nadir was defined as the consumption of wine, non-wine, coffee and tea with the lowest estimated hazard ratio (HR) over the range from 0 to the 99 % quantile of consumption and the HR at the nadir was set to 1 to simplify presentations and comparisons. HR with pointwise 95 % CI are shown for all mortality analyses. In all analyses, HR^0^ reflects HR in non-consumers of wine, non-wine, coffee or tea relative to the HR at the nadir. If both p^lin^ and p^non-lin^ were non-significant, no further interpretation of HR^0^, other individual HR or of the nadir was performed. For cause-specific mortality, survival times of participants with other causes of death were considered censored at their date of death. The proportional hazard assumption was tested based on scaled Schoenfeld residuals and all covariates violating this assumption after Holm adjustment for multiple testing were stratified in the final models and are defined in the figure and table legends. Models were adjusted for age (quartiles), sex (all participants only), total physical activity (PA; metabolic equivalent of task-min/week: <1000, 1000 to <2000, 2000 to <4000, ≥4000 and unknown), smoking status (never, previous and current), annual household income (AHI; <18, 18 to <31, 31 to <52, 52 to <100, ≥100 k£ and unknown), ethnicity (White, Group combined of Mixed, Asian, Black, Chinese and Other) and overall health rating (OHR; poor, fair, good and excellent). Since percentage body fat is also an independent and significant predictor of all-cause mortality (data not shown), all analyses were adjusted for this covariate (quartiles) as well. A *P* value of < 0·05 was considered as statistically significant in all analyses.

## Results

### Baseline characteristics and deaths in UK Biobank participants

Baseline characteristics of the study population in total and depending on wine, non-wine, coffee and tea intake are summarised in Table 1. Median (Q (Quartile) 1, Q3) age of the study population was 58 (50, 63) with 50·7 % of participants being female. Median intake was 5·7 (1·4, 11·4) g alcohol/d from wine, 4·3 (0·0, 12·9) g alcohol/d from non-wine, 2·0 (0·5, 3·0) cups/d coffee and 3·0 (1·0, 5·0) cups/d tea (Table 1). Median (Q1, Q3) wine intake was similar in female (5·7 (2·9, 11·4) g alcohol/d) compared with male (5·7 (0·7, 11·4) g alcohol/d) participants (online Supplementary Table 1). Non-wine intake was lower in females (1·4 (0·0, 4·3) g alcohol/d) as compared with males (11·4 (4·3, 22·9) g alcohol/d) (online Supplementary Table 1). Furthermore, median coffee (females 2·0 (0·5, 3·0); males 2·0 (1·0, 3·0) cups/d) and tea (females and males 3·0 (1·0, 5·0)) intake was similar between both sexes (online Supplementary Table 1). Follow-up was 12·0 (11·3, 12·7) years with 4·2 million person-years.

**Table 1.** Baseline characteristics of the UK Biobank cohort* (median values and quartiles)

Parameter	Total cohort		Wine intake in g alcohol/d								Non-wine intake in g alcohol/d							
	*n* 354 386		<1 *n* 74 357		1– <8 *n* 127 737		8–<16 *n* 89 845		≥16 *n* 62 447		<1 *n* 104 546		1–<8 *n* 118 149		8– <16 *n* 58 126		≥16 *n* 73 565	
	Median	Q1, Q3	Median	Q1, Q3	Median	Q1, Q3	Median	Q1, Q3	Median	Q1, Q3	Median	Q1, Q3	Median	Q1, Q3	Median	Q1, Q3	Median	Q1, Q3
Wine intake (g alcohol/d)	5·7	1·4, 11·4	0·0	0·0, 0·0	4·3	2·9, 5·7	10·0	8·6, 12·9	21·4	17·1, 30·0	8·6	2·9, 14·3	5·7	2·9, 11·4	5·7	1·4, 12·9	2·9	0·0, 10·0
Non-wine intake (g alcohol/d)	4·3	0·0, 12·9	10·0	2·6, 25·7	2·9	0·0, 8·6	2·9	0·0, 10·0	4·3	0·0, 11·4	0·0	0·0, 0·0	2·9	2·9, 5·7	11·4	8·6, 12·9	28·6	20·0, 42·9
Coffee intake (cups/d)	2·0	0·5, 3·0	1·0	0·0, 3·0	2·0	0·5, 3·0	2·0	1·0, 3·0	2·0	1·0, 3·0	2·0	0·5, 3·0	2·0	1·0, 3·0	2·0	1·0, 3·0	2·0	0·5, 3·0
Tea intake (cups/d)	3·0	1·0, 5·0	3·0	1·0, 5·0	3·0	2·0, 5·0	3·0	1·0, 5·0	3·0	1·0, 5·0	3·0	1·0, 5·0	3·0	2·0, 5·0	3·0	1·0, 5·0	3·0	1·0, 5·0
Age (years)	58	50, 63	57	49, 63	58	50, 63	58	50, 63	57	50, 63	58	50, 63	58	50, 63	57	50, 63	57	50, 63
	*n*	%	*n*	%	*n*	%	*n*	%	*n*	%	*n*	%	*n*	%	*n*	%	*n*	%
Female	179 676	50·7	28 548	38·4	71 264	55·8	50 053	55·7	29 811	47·7	84 040	80·4	68 381	57·9	17 965	30·9	9290	12·6
Smoking status																		
Never	187 367	52·9	36 312	48·8	78 243	61·3	47 836	53·2	24 976	40·0	61 491	58·8	70 297	59·5	28 390	48·8	27 189	37·0
Previous	131 857	37·2	26 081	35·1	40 786	31·9	35 371	39·4	29 619	47·4	36 057	34·5	39 699	33·6	23 414	40·3	32 687	44·4
Current	35 162	9·9	11 964	16·1	8708	6·8	6638	7·4	7852	12·6	6998	6·7	8153	6·9	6322	10·9	13 689	18·6
AHI (k£)																		
<18	56 502	15·9	19 987	26·9	19 460	15·2	10 510	11·7	6545	10·5	15 118	14·5	16 891	14·3	9167	15·8	15 326	20·8
18–<31	76 979	21·7	18 087	24·3	28 599	22·4	18 451	20·5	11 842	19·0	22 113	21·2	25 146	21·3	12 594	21·7	17 126	23·3
31–<52	85 496	24·1	15 701	21·1	31 366	24·6	22 630	25·2	15 799	25·3	24 602	23·5	28 467	24·1	14 493	24·9	17 934	24·4
52–<100	71 907	20·3	8546	11·5	25 486	20·0	21 416	23·8	16 459	26·4	21 598	20·7	25 251	21·4	12 227	21·0	12 831	17·4
≥100	20 172	5·7	1209	1·6	6076	4·8	6572	7·3	6315	10·1	6914	6·6	7483	6·3	3200	5·5	2575	3·5
Unknown	43 330	12·2	10 872	14·6	16 750	13·1	10 266	11·4	5487	8·8	14 201	13·6	14 911	12·6	6445	11·1	7773	10·6
Ethnicity																		
White	342 689	96·7	69 987	94·1	122 976	96·3	88 179	98·1	61 547	98·6	100 224	95·9	114 063	96·5	56 585	97·3	71 817	97·6
Mixed, Asian, Black, Chinese, Other	11 697	3·3	4370	5·9	4761	3·7	1666	1·9	900	1·4	4322	4·1	4086	3·5	1541	2·7	1748	2·4
OHR																		
Excellent	63 715	18·0	8329	11·2	23 702	18·6	19 005	21·2	12 679	20·3	21 089	20·2	22 665	19·2	10 215	17·6	9746	13·2
Good	212 997	60·1	41 093	55·3	78 408	61·4	55 666	62·0	37 830	60·6	63 880	61·1	72 421	61·3	34 888	60·0	41 808	56·8
Fair	66 994	18·9	20 298	27·3	22 620	17·7	13 540	15·1	10 536	16·9	17 043	16·3	20 161	17·1	11 342	19·5	18 448	25·1
Poor	10 680	3·0	4637	6·2	3007	2·4	1634	1·8	1402	2·2	2534	2·4	2902	2·5	1681	2·9	3563	4·8
PA (MET-min/week)																		
Median	1804		1935		1773		1787		1773		1752		1739		1828		2031	
Q1, Q3	845, 3546		813, 4158		838, 3492		874, 3386		837, 3384		834, 3345		824, 3348		852, 3582		897, 4194	
Percentage body fat																		
Median	30·2		29·6		30·8		30·4		29·6		33·9		31·2		27·7		26·9	
Q1, Q3	24·7, 36·7		24·4, 36·2		24·8, 37·3		24·8, 36·6		24·5, 35·9		28·0, 39·2		25·0, 37·6		23·1, 33·8		23·0, 31·2	

Parameter	Coffee intake in cups/d										Tea intake in cups/d									
	0 *n* 66 840		0·5–1 *n* 98 765		2 *n* 71 654		3–4 *n* 78 677		>4 *n* 38 450		0 *n* 48 959		0·5–1 *n* 42 313		2 *n* 53 093		3–4 *n* 107 229		>4 *n* 102 792	
	Median	Q1, Q3	Median	Q1, Q3	Median	Q1, Q3	Median	Q1, Q3	Median	Q1, Q3	Median	Q1, Q3	Median	Q1, Q3	Median	Q1, Q3	Median	Q1, Q3	Median	Q1, Q3
Wine intake (g alcohol/d)	4·3	0·0, 10·0	5·7	1·6, 11·4	7·1	2·9, 12·9	7·1	2·9, 12·9	5·7	1·3, 12·9	5·7	1·0, 12·9	7·1	2·9, 14·3	7·1	2·9, 12·9	5·7	2·9, 11·4	5·7	1·3, 10·0
Non-wine intake (g alcohol/d)	4·3	0·0, 14·3	2·9	0·0, 11·4	4·3	0·0, 11·4	4·3	0·0, 12·9	5·7	1·0, 17·1	5·3	0·0, 14·3	4·3	0·0, 12·9	4·3	0·0, 11·4	4·3	0·0, 11·4	4·3	0·0, 14·3
Coffee intake (cups/d)	0·0	0·0, 0·0	1·0	0·5, 1·0	2·0	2·0, 2·0	3·0	3·0, 4·0	6·0	5·0, 6·0	3·0	2·0, 5·0	3·0	1·0, 4·0	2·0	1·0, 3·0	1·0	0·5, 3·0	1·0	0·0, 2·0
Tea intake (cups/d)	4·0	3·0, 6·0	4·0	2·0, 5·0	3·0	2·0, 4·0	2·0	0·5, 4·0	1·0	0·0, 3·0	0·0	0·0, 0·0	1·0	0·5, 1·0	2·0	2·0, 2·0	3·0	3·0, 4·0	6·0	5·0, 7·0
Age (years)	56	49, 62	58	51, 63	59	51, 64	58	51, 63	56	49, 62	56	49, 62	56	49, 63	58	50, 63	58	51, 63	58	51, 63
	*n*	%	*n*	%	*n*	%	*n*	%	*n*	%	*n*	%	*n*	%	*n*	%	*n*	%	*n*	%
Female	35 568	53·2	52 565	53·2	36 786	51·3	37 946	48·2	16 811	43·7	25 585	52·3	20 603	48·7	26 401	49·7	55 711	52·0	51 376	50·0
Smoking status																				
Never	36 708	54·9	55 019	55·7	38 650	53·9	40 452	51·4	16 538	43·0	24 636	50·3	22 005	52·0	28 446	53·6	58 668	54·7	53 612	52·2
Previous	23 771	35·6	36 493	36·9	26 954	37·6	30 004	38·1	14 635	38·1	17 989	36·7	15 757	37·2	20 038	37·7	40 046	37·3	38 027	37·0
Current	6361	9·5	7253	7·3	6050	8·4	8221	10·4	7277	18·9	6334	12·9	4551	10·8	4609	8·7	8515	7·9	11 153	10·9
AHI (k£)																				
<18	12 396	18·5	16 161	16·4	10 764	15·0	11 044	14·0	6137	16·0	7932	16·2	5584	13·2	7513	14·2	17 013	15·9	18 460	18·0
18–<31	14 331	21·4	21 763	22·0	15 808	22·1	16 986	21·6	8091	21·0	10 500	21·4	8417	19·9	11 173	21·0	23 944	22·3	22 945	22·3
31–<52	15 570	23·3	23 321	23·6	12 322	24·2	19 692	25·0	9591	24·9	11 941	24·4	10 536	24·9	12 897	24·3	25 735	24·0	24 387	23·7
52–<100	12 611	18·9	19 564	19·8	14 642	20·4	16 826	21·4	8264	21·5	9931	20·3	9766	23·1	11 625	21·9	21 194	19·8	19 391	18·9
≥100	3004	4·5	5612	5·7	4402	6·1	5006	6·4	2148	5·6	2680	5·5	3340	7·9	3579	6·7	5949	5·5	4624	4·5
Unknown	8928	13·4	12 344	12·5	8716	12·2	9123	11·6	4219	11·0	5975	12·2	4670	11·0	6306	11·9	13 394	12·5	12 985	12·6
Ethnicity																				
White	63 098	94·4	94 797	96·0	69 680	97·2	77 202	98·1	37 912	98·6	47 495	97·0	40 239	95·1	50 472	95·1	103 662	96·7	100 821	98·1
Mixed, Asian, Black, Chinese, Other	3742	5·6	3968	4·0	1974	2·8	1475	1·9	538	1·4	1464	3·0	2074	4·9	2621	4·9	3567	3·3	1971	1·9
OHR																				
Excellent	10 760	16·1	17 977	18·2	13 756	19·2	14 939	19·0	6283	16·3	8678	17·7	8322	19·7	10 266	19·3	19 439	18·1	17 010	16·5
Good	39 119	58·5	59 975	60·7	43 722	61·0	47 820	60·8	22 361	58·2	28 309	57·8	25 110	59·3	32 297	60·8	65 842	61·4	61 439	59·8
Fair	14 284	21·4	17 972	18·2	12 433	17·4	13 951	17·7	8354	21·7	10 079	20·6	7662	18·1	9156	17·2	19 278	18·0	20 819	20·3
Poor	2677	4·0	2841	2·9	1743	2·4	1967	2·5	1452	3·8	1893	3·9	1219	2·9	1374	2·6	2670	2·5	3524	3·4
PA (MET-min/week)																				
Median	1852		1836		1836		1760		1720		1760		1700		1782		1813		1888	
Q1, Q3	816, 3759		876, 3546		895, 3546		825, 3426		735, 3582		777, 3572		796, 3224		855, 3452		873, 3519		869, 3813	
Percentage body fat																				
Median	30·8		30·3		30·0		30·0		29·7		31·3		29·7		29·8		30·2		30·2	
Q1, Q3	25·2, 37·2		24·7, 36·6		24·5, 36·5		24·5, 36·6		24·5, 36·7		25·4, 37·7		24·4, 36·2		24·4, 36·2		24·7, 36·6		24·6, 36·7	

AHI, annual household income; MET, metabolic equivalent of task; OHR, overall health rating; PA, physical activity; Q, quartile.

*Categorical variables are presented as number (percentage) and continuous variables as median (Q1, Q3).

### Beverage intake and all-cause mortality

Overall, 7243 deaths occurred in females and 12 958 in males, that is, a total of 20 201 deaths.

#### Wine intake

In all participants, a significant U-shaped association between wine intake and all-cause mortality was detected with the nadir at 20 g alcohol/d (Fig. 1(a)). Similar findings were observed in sex-dependent analyses with the nadir for all-cause mortality at 20 g alcohol/d for both females (Fig. 1(b)) and males (Fig. 1(c)). HR^0^ was 1·07 (1·03, 1·12) in females and 1·14 (1·11, 1·17) in males (Fig. 1(b)–(c)). A significant U-shaped association between wine intake and all-cause mortality was also detected in sensitivity analyses in cohorts S1 and S2 with the nadir between 19 and 20 g alcohol/d from wine in all participants, as well as in females and males separately (online Supplementary Fig. 2). In cohort S2, HR^0^ for present non-alcohol drinkers was higher in both females (1·23 (1·20, 1·26); online Supplementary Fig. 2(d)) and males (1·20 (1·18, 1·22); Supplementary Fig. 2(f)) compared with the corresponding HR^0^ in the primary cohort (Fig. 1(b)–(c)).

#### Non-wine intake

In all participants and in females, a significant positive dose-dependent association between non-wine intake and all-cause mortality was detected with the nadir at 0 g alcohol/d (Fig. 2(a)–(b)). In males, the nadir was at 9 g alcohol/d from non-wine with dose-dependent increases seen beyond 20 g alcohol/d (Fig. 2(c)). The shape of the association between non-wine intake and all-cause mortality changed towards a J-shaped curve in the sensitivity analyses with the most pronounced alterations seen in cohort S2 (online Supplementary Fig. 3). In cohort S2, the nadir was at 14, 7 and 17 g alcohol/d in all participants, females and males, respectively (online Supplementary Fig. 3(b), (d) and (f)).

#### Coffee intake

In all participants, coffee intake and all-cause mortality were significantly associated (Fig. 3(a)). The nadir was observed at 2 cups/d coffee and HR^0^ was significantly increased at 1·06 (1·03, 1·09) (Fig. 3(a)). Association was similar in females and males; however, statistical significance was not reached in both sexes (Fig. 3(b) and (c)). Findings were similar in sensitivity analyses using cohorts S1 and S2 (data not shown).

#### Tea intake

In all participants, all-cause mortality risk continuously decreased from HR^0^ of 1·18 (1·14, 1·21) to the nadir at 4 cups/d and no significant effects at higher consumption levels (Fig. 4(a)). Similar to all participants, HR^0^ was significantly increased in both females (1·19 (1·13, 1·25)) and males (1·17 (1·12, 1·21)) compared with the HR at the nadir (females: 7, males: 4 cups/d), respectively (Fig. 4(b) and (c)). Almost identical results were obtained in sensitivity analyses in cohorts S1 and S2 (data not shown).

### Beverage intake and cancer mortality

Within all-cause mortality, 4520 cancer deaths occurred in females and 6360 in males, that is, a total of 10 880 cancer deaths. All findings concerning the association between beverage intake and cancer mortality are summarised in online Supplementary Table 2.

#### Wine intake

In all participants and in both sexes separately, wine intake was not significantly associated with cancer mortality. Findings were numerically similar in cohorts S1 and S2, with associations reaching statistical significance in the total and male S2 cohort.

#### Non-wine intake

In all participants, as well as in females and males separately, non-wine intake was significantly associated with cancer mortality in a linear way. The nadir was at 0, 0 and 3 g alcohol/d from non-wine in all participants, females and males, respectively. Significant linear associations were also observed in cohorts S1 and S2. However, the nadir was seen at higher intake levels with a most pronounced shift towards higher values in cohort S2.

#### Coffee intake

Coffee intake was not significantly associated with cancer mortality in all participants, females and males in the primary cohort, as well as in cohorts S1 and S2.

#### Tea intake

Tea consumption was significantly associated in a non-linear form with cancer mortality and HR^0^ was significantly increased in all participants and both sexes separately. The nadir was between 3 (females) and 4 (all participants, males) cups/d. Almost identical findings were observed in cohorts S1 and S2.

### Beverage intake and non-cancer mortality

Within all-cause mortality, 2723 non-cancer deaths occurred in females and 6598 in males, that is, a total of 9321 non-cancer deaths. All findings concerning the association between beverage intake and non-cancer mortality are summarised in online Supplementary Table 3.

#### Wine intake

Wine consumption was significantly associated with non-cancer mortality in a U-shaped form in all participants and both sexes separately. The nadir was between 21 (all participants, females) and 23 (males) g alcohol/d from wine. HR^0^ was significantly increased at 1·21 (1·18, 1·25) in all participants, 1·25 (1·17, 1·33) in females and 1·19 (1·15, 1·23) in males. Whereas the nadir was similar in cohorts S1 and S2, HR^0^ was numerically higher with most pronounced increases seen in cohort S2.

#### Non-wine intake

In all participants and in females, a significant positive dose-dependent association between non-wine intake and non-cancer mortality was detected with the nadir at 0 g alcohol/d. In males, HR was lowest between 0 and 20 (nadir at 12) g alcohol/d from non-wine with dose-dependent increases seen beyond 20 g alcohol/d. The shape of the association between non-wine intake and non-cancer mortality changed towards a J-shaped curve in the sensitivity analyses with the most pronounced alterations seen in cohort S2. Here, the nadir was at 17, 8 and 19 g alcohol/d and HR^0^ was significantly elevated at 1·11 (1·09, 1·13), 1·04 (1·02, 1·07) and 1·14 (1·11, 1·18) in all participants, females and males, respectively.

#### Coffee intake

Coffee intake was significantly associated with non-cancer mortality in a non-linear manner in all participants and males. The nadir was observed at 2 cups/d and HR^0^ was slightly but significantly elevated at 1·08 (1·04, 1·13) and 1·07 (1·02, 1·12). In contrast, no significant association was observed in female subjects. Similar findings were obtained in cohorts S1 and S2 with p^non-lin^ also becoming significant in females.

#### Tea intake

Tea consumption was significantly associated in a linear and non-linear way with non-cancer mortality and HR^0^ was significantly elevated in all participants and both sexes separately. The nadir was between 5 (all participants, males) and 9 (females) cups/d. Similar results were observed in cohorts S1 and S2.

### Beverage intake and CVD mortality

Within non-cancer deaths, 916 CVD deaths occurred in females and 2858 in males, that is, a total of 3774 CVD deaths. All findings concerning the association between beverage intake and CVD mortality are summarised in online Supplementary Table 4.

#### Wine intake

Wine intake was significantly associated with CVD mortality in a non-linear manner in all participants and both sexes separately. The nadir was between 19 (females) and 21 (males) g alcohol/d. HR^0^ was significantly increased at 1·22 (1·16, 1·28) in all participants, 1·31 (1·17, 1·46) in females and 1·20 (1·14, 1·26) in males. Whereas the nadir was similar in cohorts S1 and S2, HR^0^ was numerically higher with the most pronounced increases seen in cohort S2.

#### Non-wine intake

In all participants and in males, a significant positive dose-dependent association between non-wine intake and CVD mortality was detected with the nadir at 4 and 6 g alcohol/d, respectively. The shape of the association between non-wine intake and CVD mortality changed towards a J-shaped curve in the sensitivity analyses with the most pronounced alterations seen in cohort S2. Here, the nadir was at 16 and 17 g alcohol/d and HR^0^ was significantly elevated at 1·08 (1·05, 1·11) and 1·08 (1·03, 1·13) in all participants and males, respectively. In contrast, no significant association was observed in females in the primary cohort, as well as in cohorts S1 and S2.

#### Coffee intake

Coffee intake was not significantly associated with CVD mortality in all participants, females and males in the primary cohort, as well as in cohorts S1 and S2.

#### Tea intake

Tea consumption was significantly associated in a non-linear way in all participants and males, as well as in a linear negative manner in females. HR^0^ was significantly increased at 1·20 (1·12, 1·29), 1·55 (1·34, 1·78) and 1·18 (1·09, 1·28) in all participants, females and males, respectively, with the nadir ranging from 4 (males) to 10 (females) cups/d. Similar findings were obtained in cohorts S1 and S2.

## Discussion

In the present study, it is elucidated for the first time how wine, non-wine, coffee and tea intake included as continuous non-linear predictors and mutually adjusted are associated with all-cause and cause-specific mortality.

For all participants and in sex-dependent analyses (primary cohort), a significant U-shaped association is seen between wine intake and all-cause mortality with HR^0^ significantly increased as compared with the nadir at 20 g alcohol/d. A decreased risk of death for light to moderate wine intake has also been shown in studies from the USA^(45)^, Denmark^(46)^, France^(47)^ and Sweden^(48)^ comprising 128 934, 24 523, 36 250 and 1828 individuals, respectively. To the best of our knowledge, only one study so far has assessed the sex-dependent association between different intake levels of wine and all-cause mortality. In the study by Baglietto and co-workers, HR of death is lowest in the categories 1–19 and 20–39 g alcohol/d from wine in females and males, respectively^(49)^. Similar to the current findings but using a different analytical approach, Jani and co-workers demonstrate convincingly in UK Biobank participants that HR for death decreases in red wine consumers up to around 20 weekly alcohol units^(13)^. Furthermore, red wine and champagne plus white wine intake is inversely related to all-cause mortality in another study based on UK Biobank participants in categorical analyses; however, this association largely disappears in a continuous analysis^(29)^. A different analytical approach and follow-up interval might well explain the differences between the latter results and our current findings. The present study is the first to elucidate the association between wine intake and non-cancer mortality. We show a significant U-shaped association in all participants and both sexes separately with increased HR^0^ as compared with the nadir observed between 21 and 23 g alcohol/d from wine. Similar results are obtained for CVD with the nadir detected between 19 and 21 g alcohol/d from wine. Our CVD results are well in accordance with a meta-analysis comprising six cohort studies, and the lowest risk of death was found at 24 g alcohol/d from wine^(30)^.

To the best of our knowledge, no study so far has contrasted non-wine with wine consumption regarding mortality after excluding non-drinkers. In contrast to wine consumption, a positive dose-dependent relation exists in the primary cohort between non-wine intake and all-cause mortality with the nadir observed at 0, 0 and 9 g alcohol/d from non-wine in all participants, females and males, respectively. Within the non-wine category, several studies have assessed the association of beer and spirits with all-cause mortality. In agreement with our non-wine results, Schutte and co-workers demonstrate convincingly that both beer/cider and spirits intake is associated with increased all-cause mortality risk in UK Biobank participants^(29)^. Similarly, beer/cider and spirits drinkers have a significantly higher all-cause mortality risk if compared with red wine drinkers^(13)^. A lower mortality risk in drinkers of any type of wine as compared with beer or spirits drinkers is also found in an independent cohort^(45)^. In another report, HR for all-cause mortality is increased in the highest category of beer and spirits consumption in males but not females^(49)^. In agreement with our findings, non-wine consumption is positively associated with cancer mortality in a cohort from Denmark^(46)^. Interestingly, beer/cider and spirits consumption is also associated with an increased risk for incident cancer in UK Biobank participants^(29)^. To the best of our knowledge, our study is the first to show a significant positive dose-dependent association between non-wine intake and non-cancer mortality. Within non-cancer, non-wine intake is positively and dose-dependently related to CVD mortality. Interestingly, beer/cider and spirits consumption is associated with an increased risk for cardiovascular events, ischaemic heart disease and cerebrovascular disease in UK Biobank participants^(29)^. In contrast to the current findings, a J-shaped relationship with CVD mortality is apparent for beer but not spirits^(30)^. However, this meta-analysis has not consistently controlled for the impact of non-drinkers on mortality^(30)^. Combined these findings suggest that associations between wine and non-wine consumption on the one hand and different types of mortality on the other hand show opposite directions in many cases at low to moderate drinking levels causing underestimation of mortality risk when pooled into one alcohol variable.

To the best of our knowledge, no study so far has defined the impact of not controlling for never or former alcohol drinking on the association between wine or non-wine intake and mortality. In the current study, HR^0^ for wine or non-wine increases further if never drinkers (cohort S1) and all non-alcohol drinkers (cohort S2) are included in the analysis with most pronounced changes observed in cohort S2. Decreased mortality in low to moderate alcohol drinkers in cohorts S1 and S2 could be explained by a higher death risk of former and never drinkers who might have quit or not initiated alcohol consumption because of poor health^(33,34)^. These findings support the notion that the handling of non-drinkers has a major impact on mortality analyses and might explain some discrepancies between studies. Thus, two reports demonstrate convincingly that light to moderate wine consumption is significantly related to a lower risk of death from cancer^(46,47)^ in contrast to our present results in the primary cohort. However, if we perform our analysis in cohort S2 similar to the approach used in both studies^(46,47)^, we also observe a significant non-linear association between wine intake and an increased HR^0^ for cancer deaths. Furthermore, non-wine intake of up to 21 drinks/week is not associated with an increased risk of death in one study^(46)^ in contrast to the current findings in the primary cohort. However, if we perform our analysis in cohort S2 similar to the approach used in this study^(46)^, the nadir increases to 14 g alcohol/d and risk of death is not increased up to about 25 g alcohol/d from non-wine as compared with HR^0^.

For coffee intake, the nadir is observed at 2 cups/d with non-significant effects at higher consumption levels and slightly but significantly increased HR^0^ in all participants. In agreement with the present results, increased all-cause mortality risk in non-coffee drinkers is shown in previous studies^(26–28,50)^. However, the nadir varies and is somewhat higher compared with the present report, that is, between 3 and 7 cups/d^(26–28,50–53)^. Loftfield and co-workers^(53)^ also assess UK Biobank participants with differences in study results being well explained by different exclusion criteria, follow-up time and model adjustments with coffee intake included as an ordinal variable. Coffee intake is not significantly associated with cancer mortality in the present analysis in agreement with most studies^(26,54–56)^ except one^(27)^. In the current study, the nadir for non-cancer mortality is 2 cups/d coffee in all participants with no further effects at higher amounts and a significantly increased HR^0^. To the best of our knowledge, no study so far has assessed the relation between coffee intake and overall non-cancer mortality. Within non-cancer, no significant association is detected between coffee consumption and CVD mortality. However, a dose-dependent decrease in CVD mortality as suggested by previous meta-analyses^(26,27)^ cannot be ruled out since coffee consumption levels show a nadir numerically between 2 (male) and 8 (female) cups/d. Taking published and current findings into consideration, coffee is not positively related to all-cause, cancer, non-cancer and CVD mortality. A minor negative dose-dependent association remains possible.

Tea consumption shows a significant negative dose-dependent association with all-cause mortality, and HR^0^ is significantly increased compared with HR at the nadir. Results are similar in both sexes. The nadir for all-cause mortality is between 4 and 7 cups/d tea similar to published results where it is found between 2 and >5 cups/d^(20,24,57,58)^. Similar to the present findings for all-cause mortality, tea consumption is associated with decreased cancer mortality. Although the type of tea has not been recorded during baseline assessment, the majority of tea drinkers probably consumes black tea since Great Britain has one of the highest per capita black tea consumption worldwide^(59)^. It is interesting to note in this context that the intake of black but not green tea has been linked with lower cancer mortality^(20)^. To the best of our knowledge, the present study is the first to show that HR^0^ for non-cancer mortality compared with HR at the nadir (5–9 cups/d) is significantly increased in both sexes. Findings are similar for CVD in agreement with previous reports observing a negative dose-dependent association between tea and CVD mortality in both sexes^(24,57,60)^. Taking previous publications and the present findings into consideration, tea consumption is consistently and significantly associated with decreased all-cause, cancer, non-cancer and CVD mortality in both sexes with the nadir ranging from 3 to 10 cups/d and no increases in risk of death at higher doses.

Strengths of the current report include the prospective cohort design, a large sample size, thorough characterisation of UK Biobank participants, median follow-up time >10 years, as well as the wide range of wine, non-wine, coffee and tea intake included as continuous parameters. Limitations include that consumption of other important beverages such as sugar-sweetened beverages and milk-based drinks, as well as type of tea, has not been assessed during the baseline visit. Furthermore, the present results cannot be adjusted for energy intake since this parameter was not assessed during the UK Biobank baseline visit. Further potential limitations include residual confounding, as well as measurement errors in the assessment of the exposure variables, potential confounders and misclassification of cause of death. Two studies have evaluated the performance of the UK Biobank touchscreen dietary questionnaire. As shown convincingly by Bradbury *et al.* ^(61)^ and Carter *et al.* ^(62)^, the touchscreen questionnaire adequately discriminates between high and low intakes for selected food groups when compared with a 24-h dietary assessment (Oxford WebQ). The level of agreement is comparable with estimates reported between traditional 24-h recalls and food frequency questionnaires in previous studies^(61,62)^. In addition, causal mediation analysis for specific covariates, for example, percentage body fat, could not be performed since it has not been implemented in R for Cox proportional hazard regression models with covariates included as penalised cubic splines. Moreover, a ‘healthy volunteer’ selection bias might exist since the cohort is not demographically representative of the general UK population^(63)^. However, a representative population is not required to define exposure–disease relationships^(63)^.

Summarising on a population level, the current study indicates that light to moderate consumption of wine but not non-wine is associated with decreased all-cause and non-cancer mortality. Coffee consumption is not related to increased mortality and a minor negative dose-dependent association remains possible. Tea intake is associated with a consistently decreased risk of all mortality types studied in both sexes. Further prospective studies on beverage intake in relation to morbidity from cancer and non-cancer disease are necessary to provide even more definitive conclusions.

## Supplementary material

For supplementary material accompanying this paper visit https://doi.org/10.1017/S000711452200040X. click here to view supplementary material
