# Supplementary material for: Association of alcohol types, coffee and tea intake with mortality: prospective cohort study of UK Biobank participants
Source: Br J Nutr. 2022 Feb 3;129(1):115–25. doi: 10.1017/S000711452200040X (PMC9816653; doi:10.1017/S000711452200040X)
Supplement: Supplementary file 1 [file S000711452200040Xsup001.docx]

**Supplementary Table 1** Baseline characteristics of the UK Biobank cohort depending on sex^1^

| **Parameter** | **All**  **(n=354386)** | **Female**  **(n=179676)** | **Male**  **(n=174710)** |
| --- | --- | --- | --- |
| Wine intake (g/d) | 5.7 (1.4, 11.4) | 5.7 (2.9, 11.4) | 5.7 (0.7, 11.4) |
| Non-wine intake (g/d) | 4.3 (0.0, 12.9) | 1.4 (0.0, 4.3) | 11.4 (4.3, 22.9) |
| Coffee intake (cups/d) | 2.0 (0.5, 3.0) | 2.0 (0.5, 3.0) | 2.0 (1.0, 3.0) |
| Tea intake (cups/d) | 3.0 (1.0, 5.0) | 3.0 (1.0, 5.0) | 3.0 (1.0, 5.0) |
| Age (years) | 58 (50, 63) | 57 (50, 63) | 58 (50, 64) |
| Smoking status |  |  |  |
| - Never | 187367 (52.9) | 102908 (57.3) | 84459 (48.3) |
| - Previous | 131857 (37.2) | 61792 (34.4) | 70065 (40.1) |
| - Current | 35162 (9.9) | 14976 (8.3) | 20816 (11.6) |
| AHI (£) |  |  |  |
| - <18000 | 56502 (15.9) | 29534 (16.4) | 26968 (15.4) |
| - 18000-30999 | 76979 (21.7) | 39204 (21.8) | 37775 (21.6) |
| - 31000-51999 | 85496 (24.1) | 41385 (23.0) | 44111 (25.2) |
| - 52000-99999 | 71907 (20.3) | 33229 (18.5) | 38678 (22.1) |
| - ≥100000  - Unknown | 20172 (5.7)  43330 (12.2) | 9127 (5.1)  27197 (15.1) | 11045 (6.3)  16133 (9.2) |
| Ethnicity |  |  |  |
| - White | 342689 (96.7) | 174006 (96.8) | 168683 (96.6) |
| - Mixed, Asian, Black, Chinese, Other | 11697 (3.3) | 5670 (3.2) | 6027 (3.4) |
| OHR |  |  |  |
| - Excellent  - Good  - Fair  - Poor | 63715 (18.0)  212997 (60.1)  66994 (18.9)  10680 (3.0) | 34584 (19.2)  111268 (61.9)  29529 (16.4)  4295 (2.4) | 29131 (16.7)  101729 (58.2)  37465 (21.4)  6385 (3.7) |
| PA (MET-min/week) | 1804 (845, 3546) | 1764 (838, 3380) | 1862 (850, 3732) |
| Percentage body fat | 30.2 (24.7, 36.7) | 36.2 (31.6, 40.7) | 25.3 (21.5, 28.9) |

^1^Categorical variables are presented as number (percentage) and continuous variables as median (Q1, Q3). AHI, Annual household income; MET, Metabolic equivalent of task; OHR, Overall health rating; PA, Physical activity; Q, Quartile.

**Supplementary Table 2** Association of beverage intake with cancer mortality^1^

| **Beverage** | **Primary cohort** | | | | | | | | | **Cohort S1** | | | **Cohort S2** | | |
| --- | --- | --- | --- | --- | --- | --- | --- | --- | --- | --- | --- | --- | --- | --- | --- |
|  | **P^lin^ /**  **P^non-lin^** | **nadir** | **HR^0^** | **HR at intake** | | | | | | **P^lin^ /**  **P^non-lin^** | **nadir** | **HR^0^** | **P^lin^ /**  **P^non-lin^** | **nadir** | **HR^0^** |
| **Wine** |  |  |  | **10** | **20** | **30** | **40** | **50** | **60** |  |  |  |  |  |  |
| All | 0.2978 /  0.2558 | 14 | 1.05 (1.02, 1.08) | 1.00 (0.98, 1.02) | 1.00 (0.97, 1.04) | 1.02 (0.96, 1.09) | 1.04  (0.96,  1.14) | 1.06  (0.94, 1.20) | 1.08  (0.91, 1.28) | 0.2616 / 0.0600 | 14 | 1.07  (1.04,  1.10) | 0.3109 / **0.0005** | 16 | 1.11  (1.08,  1.13) |
| Female | 0.6086 / 0.7113 | 4 | 1.00  (0.95,  1.05) | 1.00  (0.97,  1.03) | 1.02  (0.96, 1.07) | 1.03  (0.93,  1.14) | 1.03  (0.88,  1.21) | 1.01  (0.79,  1.30) | 0.99  (0.69, 1.40) | 0.5727 / 0.5197 | 11 | 1.04  (1.00,  1.09) | 0.6225 / 0.1137 | 14 | 1.08 (1.04,  1.12) |
| Male | 0.3748 / 0.0841 | 17 | 1.09  (1.06,  1.13) | 1.01 (0.98,  1.04) | 1.00  (0.95,  1.05) | 1.02  (0.95,  1.10) | 1.05  (0.95,  1.17) | 1.09  (0.94,  1.26) | 1.12  (0.93,  1.36) | 0.3464 / 0.0595 | 17 | 1.10  (1.06,  1.13) | 0.3832 / **0.0035** | 17 | 1.13  (1.10,  1.16) |
| **Non-Wine** |  |  |  | **10** | **20** | **30** | **40** | **50** | **60** |  |  |  |  |  |  |
| All | **<0.0001** / 0.0610 | 0 | 1.00 (0.98, 1.02) | 1.02 (1.00, 1.04) | 1.06 (1.03, 1.10) | 1.13 (1.08, 1.18) | 1.21 (1.14, 1.28) | 1.30 (1.22, 1.39) | 1.40 (1.30, 1.52) | **<0.0001** / 0.0602 | 1 | 1.00 (0.98, 1.02) | **<0.0001** / 0.0537 | 11 | 1.02 (1.00, 1.04) |
| Female | **0.0017** / 0.1802 | 0 | 1.00 (0.98, 1.02) | 1.04 (0.99, 1.10) | 1.14 (1.05, 1.25) | 1.26 (1.10, 1.44) | 1.31 (1.08, 1.58) | 1.30 (0.99, 1.70) | 1.36 (0.97, 1.90) | **0.0020** / 0.3648 | 3 | 1.00 (0.98, 1.03) | **0.0045** / 0.5583 | 6 | 1.02 (1.00, 1.04) |
| Male | **<0.0001** / 0.0728 | 3 | 1.00 (0.96, 1.04) | 1.01 (0.99, 1.02) | 1.03 (1.00, 1.07) | 1.09 (1.05, 1.14) | 1.18  (1.11, 1.24) | 1.28 (1.20, 1.36) | 1.38 (1.28, 1.49) | **<0.0001** / 0.0530 | 6 | 1.00  (0.96, 1.04) | **<0.0001** / **0.0182** | 14 | 1.04 (1.01, 1.08) |
| **Coffee** |  |  |  | **2** | **4** | **6** | **8** | **10** | **12** |  |  |  |  |  |  |
| All | 0.3076 / 0.4262 | 2 | 1.04 (1.00, 1.07) | 1.00 (0.98, 1.02) | 1.02 (0.99, 1.06) | 1.03 (0.97, 1.10) | 1.07 (0.96, 1.18) | 1.13 (0.96, 1.32) | 1.14 (0.84, 1.54) | 0.3215 / 0.3343 | 2 | 1.04 (1.01, 1.08) | 0.2519 / 0.2044 | 2 | 1.04 (1.01, 1.08) |
| Female | 0.1309 / 0.3243 | 1 | 1.01 (0.96, 1.07) | 1.00 (0.96, 1.04) | 1.00 (0.95, 1.06) | 1.04 (0.94, 1.15) | 1.18 (1.00, 1.40) | 1.36 (1.05, 1.77) | 1.47 (0.89, 2.43) | 0.1352 / 0.2944 | 2 | 1.02 (0.97, 1.07) | 0.0973 / 0.3848 | 2 | 1.02 (0.97, 1.07) |
| Male | 0.7369 / 0.4809 | 2 | 1.05 (1.00, 1.10) | 1.00 (0.97, 1.03) | 1.04 (0.99, 1.08) | 1.03 (0.96, 1.12) | 1.01 (0.89, 1.16) | 1.03 (0.84, 1.26) | 1.01 (0.69, 1.48) | 0.7428 / 0.3688 | 2 | 1.05 (1.00, 1.10) | 0.7361 / 0.3034 | 2 | 1.06 (1.01, 1.10) |
| **Tea** |  |  |  | **2** | **4** | **6** | **8** | **10** | **12** |  |  |  |  |  |  |
| All | 0.9086 / **0.0009** | 4 | 1.12 (1.07, 1.17) | 1.02 (1.00, 1.05) | 1.00 (0.98, 1.02) | 1.02 (0.99, 1.05) | 1.05 (1.00, 1.11) | 1.09 (1.01, 1.19) | 1.12 (1.00, 1.27) | 0.9551 / **0.0003** | 4 | 1.12 (1.08, 1.17) | 0.9035 / <**0.0001** | 4 | 1.12 (1.08, 1.17) |
| Female | 0.7112 / **0.0409** | 3 | 1.13 (1.06, 1.20) | 1.02 (0.98, 1.06) | 1.00 (0.97, 1.03) | 1.01 (0.96, 1.06) | 1.03 (0.94, 1.12) | 1.08 (0.94, 1.24) | 1.12 (0.92, 1.37) | 0.7568 / **0.0089** | 3 | 1.15 (1.08, 1.22) | 0.8577 / **0.0006** | 4 | 1.14 (1.08, 1.21) |
| Male | 0.8692 / **0.0393** | 4 | 1.11 (1.05, 1.17) | 1.02 (0.99, 1.05) | 1.00 (0.97, 1.03) | 1.02 (0.98, 1.07) | 1.07 (0.99, 1.14) | 1.10 (0.99, 1.21) | 1.12 (0.97, 1.30) | 0.8167 / **0.0377** | 4 | 1.10 (1.04, 1.16) | 0.9127 / **0.0358** | 4 | 1.10 (1.05, 1.16) |

^1^Linear (p^lin^) and non-linear (p^non-lin^) p-values for associations, the nadir, as well as HRs (95% confidence intervals) at 0 g alcohol/d or cups/d (HR^0^) are given for wine, non-wine, coffee, and tea in the primary cohort, cohort S1, and cohort S2. In addition, HRs (95% confidence intervals) at defined intake levels are shown for the primary cohort. For wine and non-wine, consumption is given as g alcohol/d. For coffee and tea, intake is given as cups/d. Covariates not fulfilling the proportional hazard assumption (primary cohort – all participants: OHR; males: age, OHR; cohort S1 – all participants: OHR; males: age, OHR; cohort S2 – all participants: OHR; males: age, OHR) are stratified.

**Supplementary Table 3** Association of beverage intake with non-cancer mortality^1^

| **Beverage** | **Primary cohort** | | | | | | | | | **Cohort S1** | | | **Cohort S2** | | |
| --- | --- | --- | --- | --- | --- | --- | --- | --- | --- | --- | --- | --- | --- | --- | --- |
|  | **P^lin^ /**  **P^non-lin^** | **nadir** | **HR^0^** | **HR at intake** | | | | | | **P^lin^ /**  **P^non-lin^** | **nadir** | **HR^0^** | **P^lin^ /**  **P^non-lin^** | **nadir** | **HR^0^** |
| **Wine** |  |  |  | **10** | **20** | **30** | **40** | **50** | **60** |  |  |  |  |  |  |
| All | **0.0023** / **<0.0001** | 21 | 1.21 (1.18, 1.25) | 1.05 (1.03, 1.08) | 1.00 (0.96, 1.04) | 1.02 (0.95, 1.09) | 1.09 (0.99, 1.20) | 1.22 (1.07, 1.38) | 1.39 (1.18, 1.63) | **0.0015** / **<0.0001** | 21 | 1.27 (1.24, 1.31) | **0.0075** / **<0.0001** | 21 | 1.35 (1.31, 1.38) |
| Female | **0.0148** / **<0.0001** | 21 | 1.25 (1.17, 1.33) | 1.09 (1.05, 1.13) | 1.00 (0.93, 1.08) | 1.04 (0.91, 1.20) | 1.20 (0.98, 1.47) | 1.52 (1.15, 2.00) | 2.09 (1.47, 2.96) | **0.0103** / **<0.0001** | 20 | 1.42 (1.35, 1.49) | **0.0162** / **<0.0001** | 20 | 1.53 (1.46, 1.60) |
| Male | **0.0228** / **<0.0001** | 23 | 1.19 (1.15, 1.23) | 1.04 (1.01, 1.07) | 1.00 (0.96, 1.05) | 1.01 (0.94, 1.09) | 1.06 (0.95, 1.18) | 1.15 (0.99, 1.32) | 1.25 (1.04, 1.50) | **0.0226** / **<0.0001** | 21 | 1.21 (1.17, 1.25) | **0.0400** / **<0.0001** | 21 | 1.28 (1.24, 1.32) |
| **Non-Wine** |  |  |  | **10** | **20** | **30** | **40** | **50** | **60** |  |  |  |  |  |  |
| All | **<0.0001** / **0.0066** | 0 | 1.00 (0.98, 1.03) | 1.01 (0.99, 1.03) | 1.05 (1.01, 1.09) | 1.10 (1.05, 1.16) | 1.18 (1.12, 1.25) | 1.28 (1.20, 1.36) | 1.38 (1.29, 1.49) | **<0.0001** / **0.0026** | 10 | 1.02 (0.99, 1.04) | **<0.0001** / **<0.0001** | 17 | 1.11 (1.09, 1.13) |
| Female | **<0.0001** / **0.0190** | 0 | 1.00 (0.97, 1.03) | 1.09 (1.03, 1.16) | 1.23 (1.10, 1.36) | 1.41 (1.22, 1.64) | 1.62 (1.33, 1.98) | 1.84 (1.42, 2.39) | 2.12 (1.55, 2.90) | **<0.0001** / 0.2634 | 4 | 1.01 (0.98, 1.03) | **<0.0001** / 0.3807 | 8 | 1.04 (1.02, 1.07) |
| Male | **<0.0001** / **0.0031** | 12 | 1.02 (0.98, 1.07) | 1.00 (0.98, 1.02) | 1.01 (0.98, 1.05) | 1.06 (1.02, 1.11) | 1.14 (1.08, 1.20) | 1.24 (1.16, 1.32) | 1.35 (1.25, 1.45) | **<0.0001** / **0.0006** | 14 | 1.04 (1.00, 1.08) | **<0.0001** /  **<0.0001** | 19 | 1.14 (1.11, 1.18) |
| **Coffee** |  |  |  | **2** | **4** | **6** | **8** | **10** | **12** |  |  |  |  |  |  |
| All | 0.5153 /  **0.0058** | 2 | 1.08 (1.04, 1.13) | 1.00 (0.98, 1.03) | 1.03 (0.99, 1.07) | 1.04 (0.97, 1.11) | 1.02 (0.92, 1.14) | 1.11 (0.94, 1.30) | 1.36, 1.04, 1.79) | 0.4395 / **0.0020** | 2 | 1.09 (1.06, 1.13) | 0.4607 / **0.0014** | 2 | 1.09 (1.05, 1.12) |
| Female | 0.1246 / 0.1786 | 8 | 1.30 (1.21, 1.39) | 1.15 (1.10, 1.20) | 1.16 (1.07, 1.25) | 1.10 (0.97, 1.26) | 1.00 (0.79, 1.26) | 0.99 (0.68, 1.43) | 1.07 (0.53, 2.16) | 0.0531 / **0.0170** | 8 | 1.31 (1.24, 1.40) | 0.1052 / **0.0297** | 8 | 1.28 (1.21, 1.35) |
| Male | 0.0949 / **0.0444** | 2 | 1.07 (1.02, 1.12) | 1.00 (0.97, 1.03) | 1.04 (1.00, 1.09) | 1.07 (0.99, 1.15) | 1.08 (0.95, 1.21) | 1.18 (0.99, 1.41) | 1.46 (1.09, 1.96) | **0.0312** / 0.0562 | 2 | 1.06 (1.01, 1.10) | **0.0194** / **0.0319** | 2 | 1.07 (1.02, 1.11) |
| **Tea** |  |  |  | **2** | **4** | **6** | **8** | **10** | **12** |  |  |  |  |  |  |
| All | **0.0029** / **<0.0001** | 5 | 1.24 (1.19, 1.30) | 1.07 (1.04, 1.09) | 1.00 (0.98, 1.03) | 1.00 (0.97, 1.04) | 1.01 (0.95, 1.07) | 1.02 (0.93, 1.11) | 1.05 (0.93, 1.19) | **0.0012** / **<0.0001** | 9 | 1.25 (1.20, 1.30) | **0.0107** / <**0.0001** | 5 | 1.22 (1.18, 1.27) |
| Female | **0.0120** / **0.0005** | 9 | 1.34 (1.24, 1.46) | 1.10 (1.05, 1.15) | 1.04 (1.00, 1.08) | 1.03 (0.97, 1.10) | 1.00 (0.89, 1.13) | 1.01 (0.84, 1.22) | 1.09 (0.83, 1.41) | **0.0009** / **0.0006** | 9 | 1.41 (1.31, 1.52) | **0.0015** / **0.0016** | 9 | 1.34 (1.26, 1.44) |
| Male | **0.0424** / **<0.0001** | 5 | 1.23 (1.17, 1.30) | 1.07 (1.04, 1.10) | 1.01 (0.98, 1.03) | 1.00 (0.96, 1.05) | 1.02 (0.95, 1.09) | 1.03 (0.93, 1.14) | 1.05 (0.91, 1.21) | 0.0765 / **<0.0001** | 5 | 1.21 (1.15, 1.28) | 0.3020 / **<0.0001** | 5 | 1.21 (1.15, 1.27) |

^1^Linear (p^lin^) and non-linear (p^non-lin^) p-values for associations, the nadir, as well as HRs (95% confidence intervals) at 0 g alcohol/d or cups/d (HR^0^) are given for wine, non-wine, coffee, and tea in the primary cohort, cohort S1, and cohort S2. In addition, HRs (95% confidence intervals) at defined intake levels are shown for the primary cohort. For wine and non-wine, consumption is given as g alcohol/d. For coffee and tea, intake is given as cups/d. Covariates not fulfilling the proportional hazard assumption (primary cohort – all participants: age, OHR, percentage body fat, sex, smoking status; females: age, percentage body fat; males: age, OHR, percentage body fat, smoking status; cohort S1 – all participants: age, OHR, percentage body fat, sex, smoking status; females: age, percentage body fat; males: age, OHR, percentage body fat, smoking status; cohort S2 – all participants: age, OHR, PA, percentage body fat, sex, smoking status; females: age, percentage body fat, smoking status; males: age, OHR, percentage body fat, smoking status) are stratified.

**Supplementary Table 4** Association of beverage intake with CVD mortality^1^

| **Beverage** | **Primary cohort** | | | | | | | | | **Cohort S1** | | | **Cohort S2** | | |
| --- | --- | --- | --- | --- | --- | --- | --- | --- | --- | --- | --- | --- | --- | --- | --- |
|  | **P^lin^ /**  **P^non-lin^** | **nadir** | **HR^0^** | **HR at intake** | | | | | | **P^lin^ /**  **P^non-lin^** | **nadir** | **HR^0^** | **P^lin^ /**  **P^non-lin^** | **nadir** | **HR^0^** |
| **Wine** |  |  |  | **10** | **20** | **30** | **40** | **50** | **60** |  |  |  |  |  |  |
| All | 0.7612 /  **0.0004** | 20 | 1.22 (1.16, 1.28) | 1.05 (1.01, 1.08) | 1.00 (0.94, 1.06) | 1.03 (0.93,  1.15) | 1.09 (0.93, 1.27) | 1.12 (0.91, 1.38) | 1.14 (0.86, 1.50) | 0.6862 / **<0.0001** | 19 | 1.28 (1.22, 1.33) | 0.7552 / **<0.0001** | 19 | 1.32 (1.27, 1.37) |
| Female | 0.4298 / **0.0439** | 19 | 1.31 (1.17, 1.46) | 1.09 (1.02, 1.16) | 1.00 (0.88, 1.15) | 1.13 (0.88, 1.45) | 1.36 (0.95, 1.96) | 1.63 (0.97, 2.74) | 1.95 (0.99, 3.83) | 0.3496 / **0.0003** | 19 | 1.50 (1.36, 1.64) | 0.3235 / **<0.0001** | 19 | 1.51 (1.40, 1.64) |
| Male | 0.9431 / **0.0031** | 21 | 1.20 (1.14 1.26) | 1.04 (1.00, 1.08) | 1.00 (0.93, 1.08) | 1.01 (0.90, 1.14) | 1.03 (0.87, 1.22) | 1.04 (0.83, 1.30) | 1.02 (0.76, 1.39) | 0.9504 / **0.0011** | 21 | 1.21 (1.15, 1.27) | 0.9580 / **<0.0001** | 20 | 1.25 (1.19, 1.30) |
| **Non-Wine** |  |  |  | **10** | **20** | **30** | **40** | **50** | **60** |  |  |  |  |  |  |
| All | **<0.0001** / 0.5215 | 4 | 1.00 (0.96, 1.04) | 1.00 (0.97, 1.04) | 1.03 (0.97, 1.09) | 1.08 (1.01, 1.16) | 1.15 (1.05, 1.25) | 1.23 (1.11, 1.35) | 1.30 (1.16, 1.46) | **<0.0001** / 0.4402 | 12 | 1.03 (0.99, 1.06) | **<0.0001** / 0.1075 | 16 | 1.08 (1.05, 1.11) |
| Female | 0.3146 / 0.8300 | 9 | 1.03 (0.97, 1.09) | 1.00 (0.89, 1.12) | 1.05 (0.86, 1.28) | 1.18 (0.88, 1.59) | 1.30 (0.87, 1.94) | 1.34 (0.76, 2.35) | 1.33 (0.65, 2.71) | 0.3545 / 0.5315 | 13 | 1.13 (1.08, 1.18) | 0.3239 / 0.4776 | 13 | 1.14 (1.09, 1.19) |
| Male | **<0.0001** / 0.4663 | 6 | 1.00 (0.94, 1.07) | 1.00 (0.98, 1.03) | 1.02 (0.98, 1.07) | 1.07 (1.01, 1.14) | 1.14 (1.06, 1.24) | 1.23 (1.12, 1.35) | 1.32 (1.18, 1.47) | **<0.0001** / 0.4045 | 11 | 1.02 (0.96, 1.08) | **<0.0001** / 0.1224 | 17 | 1.08 (1.03, 1.13) |
| **Coffee** |  |  |  | **2** | **4** | **6** | **8** | **10** | **12** |  |  |  |  |  |  |
| All | 0.4841 / 0.2896 | 2 | 1.07 (1.01, 1.14) | 1.00 (0.96, 1.04) | 1.03 (0.97, 1.10) | 1.08 (0.98, 1.19) | 1.07 (0.90, 1.26) | 1.13 (0.88, 1.46) | 1.27 (0.81, 1.98) | 0.3571 / 0.1904 | 2 | 1.08 (1.02, 1.14) | 0.3173 / 0.3403 | 2 | 1.06 (1.00, 1.11) |
| Female | 0.1988 / 0.2331 | 8 | 1.35 (1.19, 1.52) | 1.11 (1.03, 1.21) | 1.09 (0.95, 1.24) | 1.07 (0.85, 1.35) | 1.00 (0.67, 1.50) | 0.97 (0.48, 1.93) | 0.80 (0.20, 3.26) | 0.3151 / 0.0731 | 3 | 1.26 (1.13, 1.40) | 0.5401 / 0.3851 | 8 | 1.21 (1.10, 1.33) |
| Male | 0.1376 / 0.6148 | 2 | 1.03 (0.96, 1.11) | 1.00 (0.96, 1.05) | 1.05 (0.98, 1.12) | 1.11 (1.00, 1.24) | 1.11 (0.93, 1.34) | 1.18 (0.90, 1.55) | 1.35 (0.85, 2.16) | 0.0872 / 0.5759 | 2 | 1.02 (0.95, 1.09) | 0.0941 / 0.5722 | 2 | 1.02 (0.96, 1.09) |
| **Tea** |  |  |  | **2** | **4** | **6** | **8** | **10** | **12** |  |  |  |  |  |  |
| All | 0.3439 / **0.0016** | 8 | 1.20 (1.12, 1.29) | 1.06 (1.02, 1.10) | 1.01 (0.98, 1.05) | 1.01 (0.96, 1.07) | 1.00 (0.91, 1.10) | 1.01 (0.88, 1.16) | 1.08 (0.89, 1.30) | 0.2289 / **0.0011** | 9 | 1.22 (1.14, 1.30) | 0.2187 / **0.0003** | 4 | 1.21 (1.13, 1.28) |
| Female | **0.0370** / 0.3050 | 10 | 1.55 (1.34, 1.78) | 1.35 (1.25, 1.46) | 1.29 (1.20, 1.39) | 1.20 (1.07, 1.35) | 1.05 (0.86, 1.30) | 1.00 (0.72, 1.39) | 1.07 (0.67, 1.73) | **0.0150** / 0.2592 | 10 | 1.63 (1.44, 1.86) | **0.0371** / 0.3498 | 10 | 1.38 (1.23, 1.55) |
| Male | 0.9355 / **0.0055** | 4 | 1.18 (1.09, 1.28) | 1.04 (1.00, 1.09) | 1.00 (0.96, 1.04) | 1.02 (0.96, 1.08) | 1.04 (0.94, 1.15) | 1.07 (0.92, 1.24) | 1.13 (0.92, 1.40) | 0.9382 / **0.0036** | 4 | 1.19 (1.10, 1.29) | 0.9312 / **0.0007** | 4 | 1.21 (1.13, 1.31) |

^1^Linear (p^lin^) and non-linear (p^non-lin^) p-values for associations, the nadir, as well as HRs (95% confidence intervals) at 0 g alcohol/d or cups/d (HR^0^) are given for wine, non-wine, coffee, and tea in the primary cohort, cohort S1, and cohort S2. In addition, HRs (95% confidence intervals) at defined intake levels are shown for the primary cohort. For wine and non-wine, consumption is given as g alcohol/d. For coffee and tea, intake is given as cups/d. Covariates not fulfilling the proportional hazard assumption (primary cohort – all participants: age, percentage body fat, sex; cohort S1 – all participants: age, percentage body fat, sex; cohort S2 – all participants: age, percentage body fat, sex, smoking status; males: OHR) are stratified.

**Supplementary Figure legends**

**Supplementary Figure 1**

Venn diagram depicting number of participants excluded by six exclusion criteria.

**Supplementary Figure 2**

Association of wine intake (g alcohol/d) in (a), (b) the total cohort, (c), (d) females, and (e), (f) males with all-cause mortality in (a), (c), (e) cohort S1 and (b), (d), (f) cohort S2. Data are adjusted for sex (all participants only), age, AHI, ethnicity, OHR, PA, percentage body fat, and smoking status. Additionally, wine, non-wine, coffee, and tea intake are mutually adjusted (e.g., wine intake is additionally adjusted for non-wine, coffee and tea intake) as summarized in the Methods section. Covariates not fulfilling the proportional hazard assumption (cohort S1 – all participants: age, ethnicity, OHR, percentage body fat; females: age; males: age, OHR, percentage body fat; cohort S2 – all participants: age, AHI, ethnicity, OHR, percentage body fat; females: age, AHI, ethnicity, percentage body fat; males: age, OHR, percentage body fat) are stratified. The nadir is indicated in grey (total cohort), red (female), and blue (male). HR, Hazard ratio.

**Supplementary Figure 3**

Association of non-wine intake (g alcohol/d) in (a), (b) the total cohort, (c), (d) females, and (e), (f) males with all-cause mortality in (a), (c), (e) cohort S1 and (b), (d), (f) cohort S2. Data are adjusted and presented as indicated in Supplementary Figure 2.
